# A Codimension-2 Bifurcation Controlling Endogenous Bursting Activity and Pulse-Triggered Responses of a Neuron Model

**DOI:** 10.1371/journal.pone.0085451

**Published:** 2014-01-31

**Authors:** William H. Barnett, Gennady S. Cymbalyuk

**Affiliations:** Neuroscience Institute, Georgia State University, Atlanta, Georgia, United States of America; The University of Plymouth, United Kingdom

## Abstract

The dynamics of individual neurons are crucial for producing functional activity in neuronal networks. An open question is how temporal characteristics can be controlled in bursting activity and in transient neuronal responses to synaptic input. Bifurcation theory provides a framework to discover generic mechanisms addressing this question. We present a family of mechanisms organized around a global codimension-2 bifurcation. The cornerstone bifurcation is located at the intersection of the border between bursting and spiking and the border between bursting and silence. These borders correspond to the blue sky catastrophe bifurcation and the saddle-node bifurcation on an invariant circle (SNIC) curves, respectively. The cornerstone bifurcation satisfies the conditions for both the blue sky catastrophe and SNIC. The burst duration and interburst interval increase as the inverse of the square root of the difference between the corresponding bifurcation parameter and its bifurcation value. For a given set of burst duration and interburst interval, one can find the parameter values supporting these temporal characteristics. The cornerstone bifurcation also determines the responses of silent and spiking neurons. In a silent neuron with parameters close to the SNIC, a pulse of current triggers a single burst. In a spiking neuron with parameters close to the blue sky catastrophe, a pulse of current temporarily silences the neuron. These responses are stereotypical: the durations of the transient intervals–the duration of the burst and the duration of latency to spiking–are governed by the inverse-square-root laws. The mechanisms described here could be used to coordinate neuromuscular control in central pattern generators. As proof of principle, we construct small networks that control metachronal-wave motor pattern exhibited in locomotion. This pattern is determined by the phase relations of bursting neurons in a simple central pattern generator modeled by a chain of oscillators.

## Introduction

The dynamics of individual neurons are crucial for the functionality of neuronal networks. Precise timing and reliability of temporal responses are critical for memory, pattern recognition, and especially motor control [1]–[10]. Functional bursting activity, latency to spiking, and transient oscillatory activity are necessary components determining reliability and precise timing. For example, transient spiking activity is critical for information representation and memory [2], [3], [11], [12]; precise control over delay to firing after inhibition is important for the detection of temporal patterns [4]–[6]; and bursting activity is crucial for rhythmic motor functions including respiration, locomotion, and neurogenic cardiac systems [7], [8].

Rhythmic motor functions are executed by precisely coordinated oscillatory patterns of contracting muscles. These functions require flexibility of rhythmic patterns to cope with environmental conditions such as temperature, load, or the demand for speed of locomotion. Accordingly, some rhythmic behaviors scale their pattern, maintaining phase relations across a wide range of periods, for example, according to different speeds of locomotion. Examples of pattern scaling behaviors include the pyloric rhythm in Crustacea, crayfish swimmeret beating, the leech heartbeat, leech swimming, lamprey swimming, and crawling of the *Drosophila* larvae [8]–[10], [13]–[24].

Rhythmic behaviors are controlled by devoted oscillatory neuronal networks: central pattern generators (CPGs) [7], [8], [15], [17], [25]–[29]. CPGs adjust their patterns according to motor tasks, sensory feedback, and environmental conditions [14], [15], [22], [29]. They are comprised of neurons from a spectrum of endogenous properties varying from tonic spiking through bursting to silent neurons [7], [25]–[29]. These neurons could be sensitive to neuromodulatory tone, descending tonic drive, and phasic sensory feedback [25]–[27], [29]. Neurons like conditional oscillators could be found in either tonic spiking, bursting, or silent regimes, when decoupled from a circuit [25]–[27], [29]. Major open questions are concerned with determining the key cellular properties which are characteristic for neurons in CPGs. Are there organizing principles of cellular dynamics which allow neurons to produce precise patterns in an orchestrated fashion? Are there mechanisms which coordinate dynamic properties of neurons to accomplish adaptations of motor behavior to continuously changing conditions?

Bifurcation theory explains how dynamical systems like neurons precisely change their dynamics in response to the variation of a controlling parameter like neuromodulatory tone or descending drive [1]–[3], [6], [11], [12], [30]–[51]. A variety of network mechanisms producing specific temporal patterns of activity have been previously studied [1], [10], [18], [31], [34], [41], [52]–[60].

Our study emphasizes the dynamics of single neurons as an organizing principle underlying pattern formation. We present a global codimension-2 bifurcation and assert that this so-called cornerstone bifurcation can precisely control the regulation of temporal characteristics in periodic and transient neuronal activity. We suggest that this bifurcation generates a family of cellular mechanisms which can answer the aforementioned questions. These mechanisms also explain the operation of conditional oscillators in parameter space near a functional bursting regime.

In neurons, control over regimes of activity is ubiquitously executed through neuromodulation. Potassium currents and hyperpolarization-activated currents are common targets for neuromodulation [61]–[68]. Among the usual targets of neuromodulation are the kinetic parameters controlling the voltage dependence of conductances of ionic currents such as a non-inactivating potassium current, 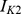, and a hyperpolarization-activated current, 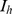. 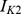 is an outward current that is activated during the burst and creates a mechanism controlling termination of the burst. To serve this role, the activation variable of this current must be significantly slower than the duration of a single spike. On the other hand, 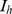 is an inward current that is activated during the silent phase of bursting activity, and it creates a mechanism controlling the duration of the silent phase. The voltage of half-activation of 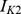, 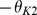, could control burst duration, and the voltage of half-activation of 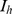, 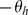, could control the interburst interval. Here, we present a model in which the dependence of burst duration and interburst interval on 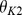 and 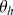 can be quantitatively described by inverse-square-root laws.

Biophysically accurate neuronal models allow the utilization of well developed techniques from bifurcation theory. In a model, bifurcations can predict the dependence of temporal characteristics of oscillatory regimes near the bifurcation [11], [12], [42], [43], [48]. In type I spiking neuronal dynamics, a saddle-node bifurcation on an invariant circle (SNIC) describes a transition from tonic spiking into silence; the interspike interval obeys the inverse-square-root law imposed by the bifurcation. The period of spiking grows proportionally to 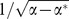 where 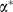 is the bifurcation value of the parameter 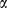 and 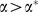. In our case, the bifurcation parameters are either 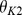 or 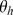. The blue sky catastrophe, a special case of the saddle-node bifurcation for orbits, imposes the same inverse-square-root law on burst duration [45], [69]. A saddle-node bifurcation for periodic orbits can control the transition from bursting into tonic spiking [11], [12], [45], [70]. When the criteria for the SNIC and the blue sky catastrophe are simultaneously satisfied, a global bifurcation of codimension-2 occurs [69], [70]. By coregulating these currents, we reveal the cornerstone bifurcation and how the bifurcation parameters control burst duration and interburst interval, latency to spiking in the response of a spiking neuron to inhibition, duration of a burst elicited by stimulation of a silent neuron, and multistability of spiking and silence. We apply these mechanisms to construct simple proof of principle models of CPGs. One of these mechanisms describes the coregulation of two biophysical parameters in the vicinity of the cornerstone bifurcation such that the duty cycle of bursting in a single cell is maintained across a wide range of periods. Connected into a chain, such oscillators self-organize their activity into a metachronal wave pattern, which is preserved against variation in period. We present this model in regard to crawling behavior in *Drosophila* larvae.

## Results

### Regimes of Activity

We developed a generic low-dimensional Hodgkin-Huxley type neuronal model stemming from a model of the leech heart interneuron under certain pharmacological conditions [44], [45]. In order to reduce the mathematical complexity of the system, we simulated the activity of the interneuron in bath with Co^2+^ and 4-aminopyridine (4-AP). Application of Co^2+^ blocks Ca^2+^ currents and the synaptic current. Application of 4-AP blocks most of the K^+^ currents. The remaining currents in this model are the leak current, the non-inactivating potassium current, 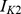, the fast sodium current, 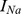, and a constant polarizing current. Our model also includes the hyperpolarization-activated current, 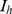, which is present in this pharmacological scenario but has not been previously represented in our reduced models. Intracellular recordings in these conditions show slow seizure-like oscillations with periods that are tens of seconds long [71], [72]. The slow variable in this system is the activation of 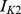, 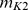; its time constant was 2 s. As such, the temporal characteristics of these variables are instrumental in the dynamics of bursting activity. By systematically manipulating parameters that determine the dynamics of the slow variables, we observed the range of bursting activity and bifurcations between qualitatively distinct regimes of neuronal activity. We investigated the effects of changes to the kinetics of 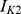 and 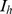.

**Figure 1:**
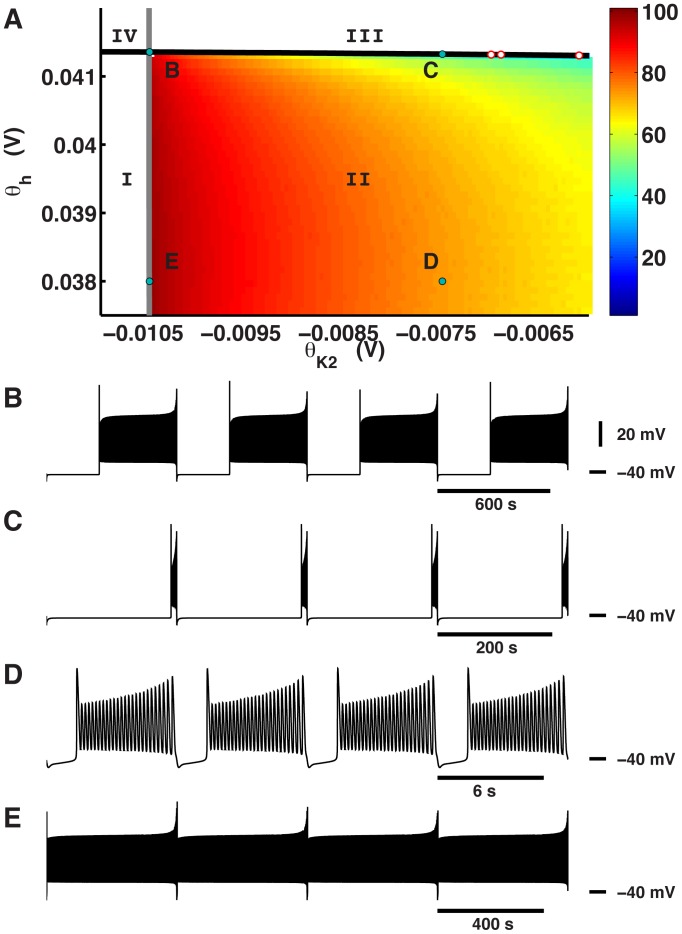
Tonic spiking, bursting, and silence are mapped onto the (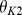, 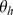) bifurcation diagram. (A) Tonic spiking, bursting, silence, and multistability of tonic spiking and silence are supported in the corresponding parameter regions labeled 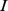, 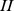, 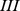, and 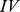. Bursting is described by duty cycle, which is the ratio of burst duration to cycle period. Duty cycle is represented as a color map from 0 to 100%. The three empty red circles mark sample parameters sets with duty cycle 10%. The grey curve indicates the position of the saddle-node bifurcation for periodic orbits. The black curve indicates the position of the saddle-node bifurcation for equilibria. Examples of waveforms of bursting activity at four different parameter sets (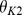, 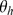): (B) (−0.0105 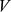, 0.0413564925 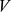), (C) (−0.0075 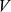, 0.041326 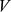), (D) (−0.0075 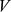, 0.038 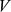), and (E) (−0.0105 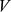, 0.038 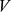). The point at (B) is near but not on the codimension-2 bifurcation point.

The parameters 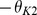 and 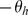 represent the voltages of half-activation of the variables 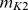 and 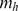. We conducted an empirical investigation of the model by systematically varying 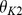 and 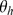. The model exhibited a variety of regimes. Silence and tonic spiking corresponded to equilibria and periodic orbits. Bursting activity was either periodic or weakly chaotic. For low values of 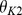 and 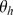 (region 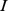), the model exhibited tonic spiking (Fig. 1A). For high values of 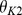 and low values of 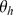 (region 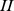), the model exhibited bursting activity (Fig. 1A). For high values of 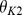 and 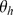 (region 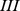), the model was silent (Fig. 1A). For low values of 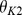 and high values of 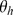 (region 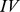), the model exhibited bistability of tonic spiking and silence (Fig. 1A).

We determined the boundaries between bursting and tonic spiking and between bursting and silence by integrating the system over a range of values for 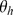 and 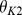. Samples varied from one another in the burst duration and the interburst interval (Table 1). The transition from bursting to tonic spiking occurred near 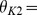
−0.0105 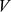 almost independently of 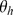 (Fig. 1A). The transition from bursting to silence occurred near 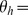 0.0413 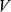 with weak dependence on 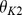 (Fig. 1A). As the parameters were changed to approach the transition from bursting to tonic spiking or from bursting to silence, the burst duration or interburst interval increased, respectively. We described the changes in the bursting wave form by duty cycle: the ratio of burst duration to cycle period. The duty cycle generally increased as 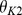 or 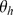 decreased (Fig. 1A).

**Table 1:** Temporal characteristics of bursting for different parameter values in region 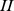.

Figure 1:	(B)	(C)	(D)	(E)
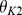 ( 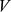 ):	−0.0105	−0.0075	−0.0075	−0.0105
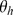 ( 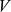 ):	0.0413564925	0.041326	0.038	0.038
burst duration (s):	412.0	9.8	5.4	488.3
interburst interval (s):	281.6	217.5	2.0	1.9

### The Inverse-Square-Root Laws Control Bursting Activity

We associated saddle-node bifurcations of equilibria and periodic orbits with the transitions between different regimes. The transitions between 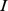 and 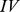 and between 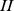 and 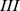 coincided with the saddle-node bifurcation for equilibria (Fig. 1A, black curve). The borders between regions 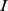 and 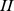 and between regions 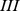 and 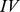 coincided with the saddle-node bifurcation for periodic orbits (Fig. 1A, grey curve). The location of the codimension-2 bifurcation was interpolated as the intersection of the curves representing the saddle-node bifurcation for equilibria and the saddle-node bifurcation for periodic orbits at (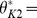
−0.010505 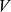, 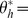 0.041356538 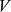).

We show that the saddle-node bifurcations along the border between 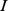 and 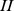 and between 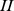 and 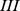 are a blue sky catastrophe and a SNIC, respectively, through a series of curve fits and analysis of slow motion (Text S1; Text S2). We investigated temporal characteristics of bursting near these bifurcation curves (Text S1; Fig. S1). The system was directly integrated for a series of parameter values approaching the bifurcation values. As 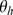 approached the saddle-node bifurcation for equilibria, the interburst interval grew in a fashion asymptotic to the parameter value of bifurcation (Fig. S1A–B). The parameter 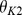 was similarly varied so as to approach its critical value for the saddle-node bifurcation for periodic orbits. Samples of bursting activity for values of 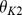 successively closer to the bifurcation value showed an asymptotic increase in the burst duration (Fig. S1C–D). The curve fits of the burst duration and interburst interval conformed to the 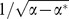 temporal law at multiple locations along the bifurcation curves (Text S1). We also confirmed these results for the value of the time constant of activation of the potassium current 0.9 s used in the [45].

### The Inverse-Square-Root Laws of Pulse Triggered Responses

**Figure 2:**
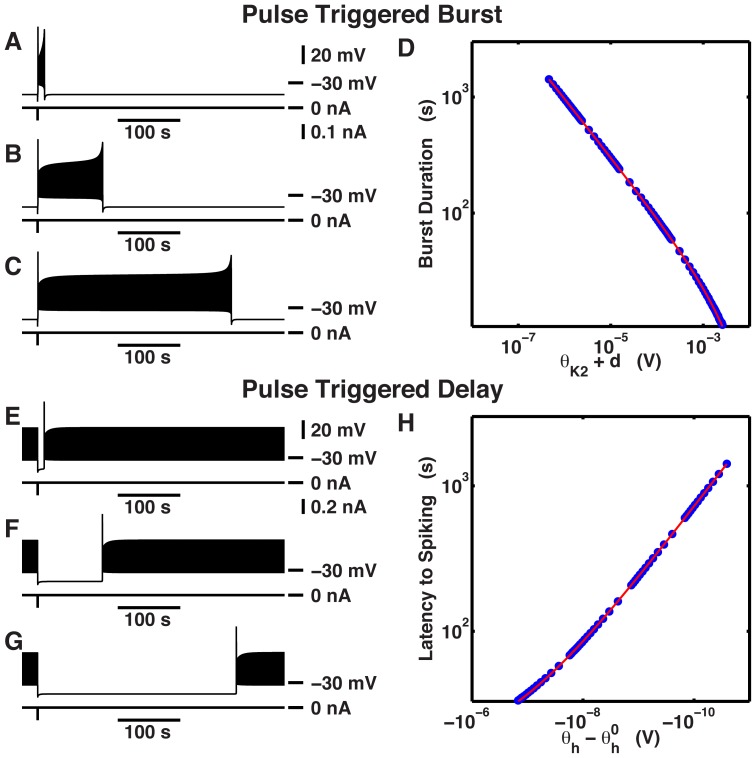
The inverse-square-root laws in transient responses triggered by a pulse of current for parameter values that support silent (region 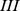) and tonic spiking (region 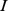) regimes. (A–D) An individual burst was triggered by a hyperpolarizing pulse of injected current. Pulses of current were 0.03 s in duration and 0.1 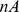 in amplitude. The duration of individual bursts were (A) (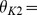
−0.0077 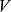, 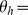 0.0415 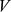) 10.327403 s, (B) (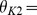
−0.01043 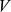, 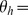 0.0415 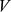) 103.48097 s, and (C) (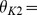
−0.010496 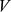, 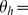 0.0415 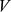) 309.27622 s. (D) The log-log graph of burst duration of individual bursts plotted against 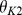. The blue dots correspond to the burst duration measured at the respective values of 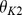. The red curve is the graph of the curve fitted in the form 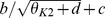. Coefficients of the curve fit are 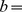 0.97239439, 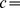
−8.48809474, and 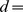 0.01050536. (E–H) Latency to spiking was shown by administering an individual pulse of injected current. Pulses were 0.03 s in duration and 0.2 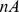 in amplitude. The delays shown here are (E) (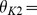
−0.0107 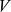, 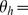 0.04134 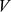) 10.287 s, (F) (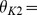
−0.0107 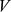, 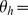 0.041358041 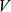) 103.378 s, and (G) (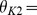
−0.0107 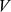, 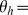 0.0413580468 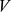) 317.679 s. (H) latency to spiking for sampled parameter values (blue dots) and the graph of the curve fitted to 
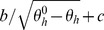
 (red curve) were plotted in log-log scale against the sampled values of 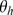. Coefficients of the fitted curve were 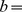 0.00709613 and 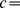 14.47354286. The parameter 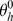 was 0.04135804734566 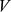.

For parameter values in regions 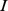 and 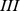 (Fig. 1), tonic spiking and silence were attracting regimes. However, a perturbation from the tonic spiking regime in 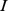 or the rest state in 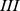 triggered transient activity that shared the inverse-square-root laws with the corresponding characteristics of periodic bursting in region 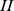 (Fig. 2). For example, activity at parameter values in region 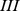 was quiescent, but a brief hyperpolarizing pulse of appropriate duration and amplitude triggered a single burst (Fig. 2 A–C). These individual bursts closely resembled the characteristic waveforms of bursting activity observed in Region 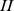. We investigated this parameter space by observing pulse-triggered bursts at parameter values such that 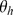 was fixed at 0.0415 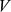 and 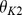 approached the value for the saddle-node bifurcation for periodic orbits at the border between regions 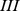 and 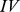. As 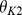 approached the value of the bifurcation, the burst duration of pulse triggered bursts grew (Table 2). Even though there was no closed periodic orbit in region 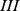, the phase point slowed down as it passed near the ghost of the saddle-node periodic orbit. We performed a curve fit of this data to the function 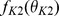 (Eq. 1 in Text S1) (Fig. 2D).

**Table 2:** Measure of burst duration and latency to spiking in response to inhibition from Figure 2 (A−C,E−G).

Figure 2	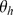 ( 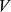 )	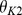 ( 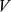 )	burst duration (s)	latency to spiking (s)
(A)	0.0415	−0.0077	10.327403	NA
(B)	0.0415	−0.01043	103.48097	NA
(C)	0.0415	−0.010496	309.27622	NA
(E)	0.04134	−0.0107	NA	10.287
(F)	0.041358041	−0.0107	NA	103.378
(G)	0.0413580468	−0.0107	NA	317.679

We carried out a similar analysis for the border of the transition from regions 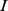 to 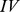. When tonic spiking activity was perturbed with a hyperpolarizing pulse, the system spent some time in a transient hyperpolarized silent state before returning to spiking activity. The trajectory during this time interval resembled the trajectory during the interburst interval of an endogenously bursting cell. We fixed 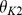 at −0.0107 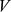, and we sampled region 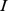 for values of 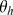 close to the saddle-node bifurcation for equilibria (Fig. 2E–G). As 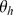 grew, the latency to spiking grew (Table 2). As the parameter 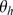 took values close to the saddle-node bifurcation for equilibria, the phase point spent more and more time near the ghost of the saddle-node equilibrium. We quantified the dependence of the latency to spiking on 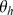 by performing a curve fit to the function 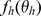 (Eq. 2 in Text S1) (Fig. 2H).

### The Manifolds of Slow Motion

This model exhibit bursting of square-wave type. The bursting phase is controlled by the activation of 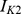. Over the course of the burst, 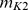 incrementally grows with each spike. Vice versa, 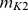 controls the shape of each spike. The shape of each spike depends on the value of 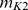, and the next increment of 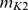 depends on the shape of the spike. To account for this mutual interaction and to formalize the progression of the burst towards termination, we used slow-fast decomposition. This technique allows us to describe this process in terms of the dynamics of one variable: the slow variable.

We considered the model as a slow-fast system. The variable 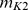 was the slow subsystem, and 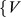, 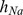, 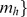 composed the fast subsystem. The magnitude of 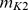 controls rest states (equilibria) and spiking (periodic orbits) of the fast subsystem. These equilibria and periodic orbits are located on manifolds that could be determined by decoupling the slow-fast system and reintroducing the slow variable as a parameter [35], [49]. Trajectories of the full system stay near these manifolds of slow motion [35], [49], [73]. The slow motion manifold that determines periodic orbits describes the breadth of specifications of spike shapes in the fast subsystem. The slow motion manifold that determines the silent phase describes the characteristics of equilibria of the fast subsystem. We used a more accurate approach, whereby we manipulated a parameter of the slow variable (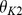) to compute the manifolds of slow motion in the full system [45]–[47], [74]. We performed slow-fast decomposition and determined the slow motion manifolds to study the structure of the codimension-2 bifurcation. We applied the Pontryagin 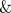 Rodygin averaging method [45]–[47], [74]. The stability of the regimes in the full system was determined by bifurcation analysis (Text S2).

**Figure 3:**
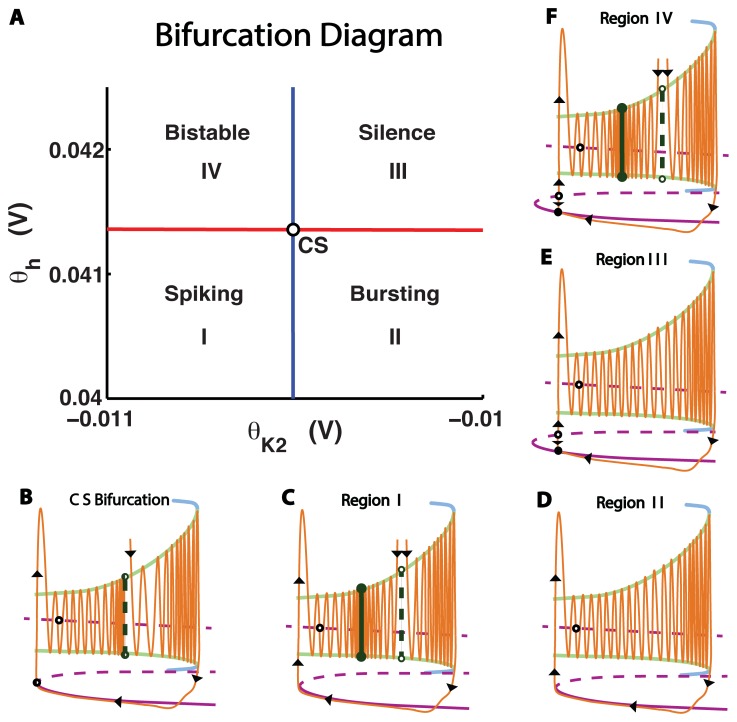
Diagram of the cornerstone bifurcation (CS). (A) The CS is located at the intersection of the saddle-node bifurcation for equilibria (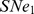; red curve) and the saddle-node bifurcation for periodic orbits (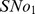; solid blue curve). The dashed blue curve is 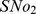, where a large amplitude stable orbit is born. The solid green curve is a period doubling bifurcation. A series of period doubling bifurcations occur between this curve and the dashed green curve, where the large amplitude regime terminates. For values of 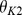 larger than where this regime terminates, we consider four adjacent regions of the parameter space. In the region marked 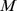, we observe only a small amplitude orbit, which corresponds to tonic spiking. In 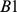, a large amplitude orbit co-exists with the small amplitude orbit. In 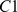, a the large amplitude orbit becomes chaotic and vanishes in a period doubling cascade. In 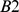, the tonic spiking regime co-exists with a stable equilibrium. In 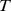, the small amplitude orbit and the stable equilibrium co-exist with a large amplitude orbit. In 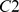, the large amplitude orbit becomes chaotic and vanishes in a period doubling cascade. (B–F) Representations of the dynamics of the system at different points in the parameter space. The orange curves represent trajectories, and the black arrows indicate the direction of motion of the phase point. The two sets of light green and blue curves represent the maximum and minimum of orbits on the slow motion manifolds for oscillations. The green and blue portions indicate the attracting and repelling segments of this manifold, respectively. The solid and dashed purple curves correspond to stable and unstable equilibria in the fast subsystem, respectively. Filled red dots represent stable equilibria, and unfilled red dots represent unstable equilibria. Solid and dashed vertical dark green lines represent stable and unstable simple periodic orbits, respectively. (B) The structure of the state space at the CS point. A saddle-node periodic orbit exists on the slow motion manifold for oscillations, and a saddle-node equilibrium exists on the slow motion manifold corresponding to the equilibria of the fast subsystem. (C) A stable periodic orbit and a saddle periodic orbit exist on the slow motion manifold for oscillations. (D) Periodic bursting is observed. The phase point moves as indicated by the black arrows in a clockwise fashion. (E) A stable equilibrium of the full system obstructs the stable segment of the equilibria of the fast subsystem. (F) Spiking co-exists with the silent regime.

The parameter space that we analyzed is partitioned by two bifurcation curves: one horizontal and one vertical (Fig. 3). The horizontal curve is a saddle-node bifurcation for equilibria (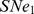 in Fig. S2). It divides the 2-D parameter space into two subspaces (red curve in Fig. 3A). At this bifurcation, a stable equilibrium disappears; the parameter space above this curve supports the stable equilibrium. The vertical solid blue curve represents a saddle-node bifurcation for periodic orbits (Fig. 3A; 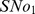 Fig. S2). At this bifurcation, a stable periodic orbit–representing tonic spiking–disappears; the parameter space to the left of this curve supports tonic spiking.

The cornerstone bifurcation, which is located at the intersection of the two bifurcation curves (red and blue solid curves), organizes bursting, spiking, and silent regimes (Fig. 3A). The bifurcations defining the borders of region 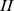 (SNIC and blue sky catastrophe) lead to qualitative changes in the vector field which eliminate periodic bursting by obstructing the passage of the phase point over one or both manifolds of slow motion (Fig. 3A).

At the codimension-2 bifurcation point, the slow motion manifold determined by the equilibria of the fast subsystem is obstructed by a saddle-node equilibrium, and the slow manifold for spiking is obstructed by a saddle-node bifurcation for periodic orbits (Fig. 3B).

Perturbations in the (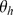, 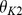) parameter space from the codimension-2 point lead to the disappearance of the saddle-node equilibrium or simple periodic orbit or cause the saddle-node orbit or equilibrium to split in two (Fig. 3C–F).

In region 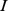, a stable orbit and a saddle orbit were found on the slow manifold for spiking (Fig. 3C). The stable periodic orbit corresponds to the tonic spiking regime, and is the only attracting regime in this region. Perturbations can reveal characteristics of the slow manifold associated with the equilibrium of the fast subsystem. The phase point can spend a significant amount of time in a quiescent state before firing resumes after the cessation of an inhibitory perturbation (Fig. 2E–G). This perturbation causes a fast transition of the phase point from a stable orbit onto the the slow-motion manifold of equilibria for the fast subsystem. The phase point followed the slow-motion manifold, and the neuron exhibited a transient quiescence. For parameter values near the border between regions 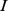 and 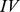, the phase point could spend a long time near the ghost of the saddle-node equilibrium before the onset of firing.

In region 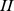, both the manifolds of slow motion are unobstructed. This parameter region corresponds to the endogenous bursting regime (Fig. 3D). During a burst, the phase point winds around the slow motion manifold for oscillations; during the interburst interval, the phase point follows the slow motion manifold for equilibria in the fast subsystem.

In region 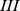, a stable equilibrium obstructs the slow manifold for equilibria of the fast subsystem (Fig. 3E). The stable equilibrium is the only attracting regime in this region. A perturbation can trigger a train of action potentials (Fig. 2A–C). During a transient burst, a fast transition occurred as the phase point moved from the stable equilibrium onto the slow manifold that controls oscillating activity in the fast subsystem. The model neuron executed a stereotyped burst as the phase point evolved across this manifold. Near the border between regions 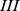 and 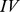, the phase point could spend a long time near the ghost of the saddle-node orbit, producing a very long transient burst.

In region 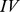, there were stable and unstable orbits as well as stable and unstable equilibria. As a result, tonic spiking co-existed with a silent regime, and perturbations could elicit switches from one regime to another (Fig. 3F).

The mechanisms controlling burst duration and interburst interval in region 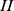 determine a scheme of two-parameter coregulation that supports bursting with a given duty cycle across a wide range of cycle periods. This scheme could explain the maintenance of phase relations in a network of coupled oscillators.

### Pattern-scaling in a Chain of Oscillators

We applied a mechanism maintaining the duty cycle of endogenously bursting activity to show scaling in a metachronal-wave locomotor pattern. We developed a basic model of a central pattern generator for crawling of larval *Drosophila*. *Drosophila* larvae crawl by means of peristaltic contractions of the body. Posterior-to-anterior waves of peristaltic contraction produce forward motion. The phase lag of motor activity in neighboring segments scales proportionally to the period so that the phase relations between activity in neighboring segments is maintained. This scaling occurs over a two-fold range of cycle periods [24]. Activity in the nerve cord in isolated preparations occurs on the time scale of tens of seconds [20], [21]. The phase delay from segment-to-segment of segmental nerve cord activity is roughly 10% of the cycle period [20]. We suggest that the scheme of coregulation controlling the duty cycle of participating neurons could present a cellular mechanism for the metachronal-wave pattern scaling. We modeled the metachronal-wave central pattern generator (CPG) as a chain of coupled oscillatory neurons.

The network was assembled from five endogenously bursting neurons from region 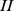 (Fig. 1A). If these neurons receive an inhibitory pulse during the interburst interval, they respond with a burst, and the activity is reset. This observation suggests that if these neurons are coupled in a chain, they will trigger burst responses one after another as though in a domino effect. We expect that this effect would be robust if the neurons were connected in a chain such that the strength of inhibitory coupling is stronger in one direction than in the other. This sequential propagation of excitation along a chain is referred to as a metachronal wave which would travel in the direction of stronger coupling. With this organization of activity, the phase delay is determined by burst duration. Thus by scaling the duty cycle, one can scale the pattern of the metachronal wave. We manipulated the network by changing the values of 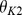 and 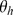 to control the burst duration and interburst interval. We coordinated these changes to maintain a constraint of 10% duty cycle within 0.1% tolerance for individual oscillators over a wide range of periods from 15 s to 85 s (Table 3). We predicted that a chain of coupled oscillators each with an intrinsic duty cycle of 10% would produce a metachronal wave with phase delay of 10% at the period of the individual oscillators. With this duty cycle, in order to produce rhythms faster than 68 s, we obtained parameter sets with values of 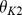 beyond the range depicted in Figure 1A. They are located so close to the SNIC that they appear to sit on the curve (Fig. 1A). We did not investigate whether the SNIC extends beyond this range, yet we were able to obtain parameter sets that supported activity with a duty cycle of 10%.

**Table 3:** Cycle period, duty cycle, and values for 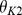 and 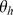. Labeled parameter sets were used to produce activity found in Figure 4.

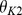 ( 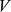 )	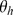 ( 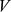 )	Period (s):	Cell	Network	Duty Cycle (%):	Cell	Network	
0.0059	0.040736		15.1	15.1		10.0	9.9	Fig. 4(C)
0.0019	0.041048		21.0	21.0		10.0	11.0	
−0.0001	0.041145		27.4	27.4		9.9	9.9	
−0.0011	0.041183		31.8	31.8		9.9	9.8	
−0.0031	0.041244		39.5	39.5		10.0	10.4	
−0.0041	0.041268		48.0	48.0		10.0	9.9	Fig. 4(B)
−0.0057	0.041298		61.8	61.8		10.0	10.3	
−0.0061	0.041306		68.2	68.1		10.0	10.3	
−0.0069	0.041318		82.3	82.1		10.0	10.0	
−0.0070	0.041319		85.3	85.1		9.9	9.9	Fig. 4(A)

All parameter sets were used to produce activity used in the analysis found in Figure 5. Average network cycle period and average network duty cycle each had coefficients of variation less than 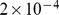 % and 4%, respectively.

**Figure 4:**
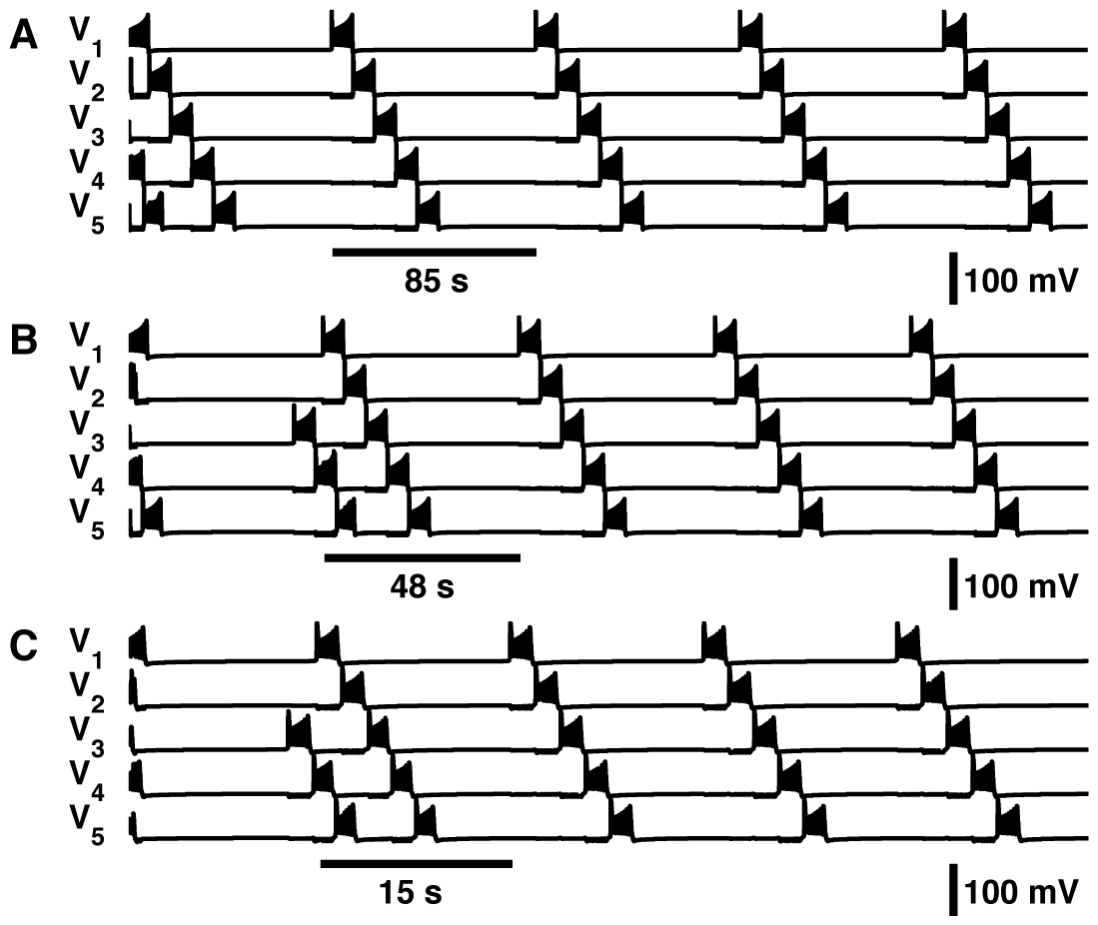
Scaling of the metachronal-wave pattern in chains of coupled endogenously bursting neurons. Neurons connected through inhibitory coupling with strongest connections to the immediate anterior neighbor. Metachronal Waves produced by three examples of the network. The parameter values for (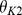, 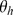) used to create these trajectories were as follows: (A) (−0.0069999 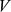, 0.041319316864014 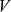), (B) (−0.0040999 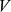, 0.041268055725098 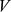), and (C) (0.005905 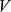, 0.04073603515625 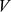).

The model is represented by a chain of identical bursting neurons with duty cycle 10% as described in Methods. To show pattern scaling, we investigated this model with parameters of individual cells varied according to our coregulation scheme defined in Table 3. Network activity self-organized into a metachronal-wave pattern following an interval of transient activity in all ten instantiations (Fig. 4). After the interval of transient activity, waves of bursts propagated along the chain of oscillators from the most posterior cell (segment 7) in the anterior direction. We quantified the time it took a network to reach a periodic state by counting the number of cycles of the posterior cell before the transient activity subsided and the propagating wave fully formed. In each instantiation of the network, the formation of the metachronal wave occurred within two cycles of the bursting neuron in segment 7.

**Figure 5:**
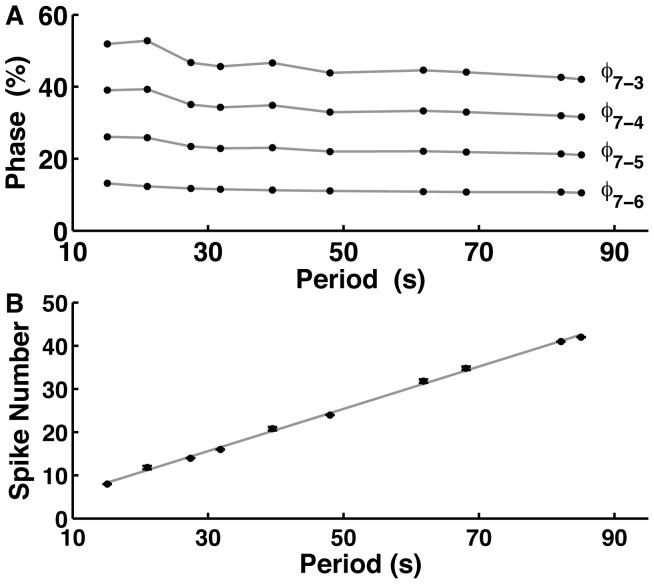
Temporal characteristics of metachronal wave pattern across a range of cycle periods. The phase relations of a metachronal-wave pattern are determined by the duty cycle of each element in a chain of inhibitory coupled bursting neurons. The parameters are changed in a coordinated fashion to support different cycle periods while the duty cycle is kept constant. (A) The phases of oscillators are portrayed relative to the oscillator in the seventh segment. (B) The average number of spikes per burst varies linearly with cycle period as 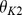 and 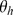 are changed. The black markers indicated average spike number with error bars. The grey line was the linear function for spike number fitted to our data: 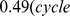 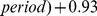.

We characterized metachronal waves in the activity of each network instantiation by the period and duty cycle of activity as well as the relative phase shift of bursting from one segment to its nearest neighbor. We compared the temporal characteristics of network bursting activity to those of activity produced by a single cell with the same values of 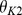 and 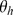 (Table 3). The period and duty cycle of bursting activity of a single cell and the network matched well; these periods differed by less than 0.1% and 3%, respectively. The disparity between the period of single-cell and network activity was greater for network instantiations with larger period. In each network instantiation, the phase shift of the metachronal wave exhibited some dependence on cycle period (Fig. 5). For parameter values that produced activity with lower periods, the phase shift tended to be greater than that predicted by the duty cycle. For example, in network instantiations with parameter values that produced activity with period 15.1 s and 21.0 s, the average relative phase between segments was 13.0% and 13.2%, respectively. For parameter values that produced activity with higher periods, the phase shift tended to be close to 10%. The instantiation of the network that produced activity with period 85.1 s had an average relative phase shift of 10.5%.

The cornerstone bifurcation controls periodicity through both burst duration and interburst interval. By coregulating 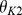 and 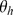, we vary the period of bursting while keeping the duty cycle fixed. As instantiations of the network become closer to the critical parameter values for the cornerstone bifurcation, the period of activity increases according to the inverse-square laws governing burst duration and interburst interval. Since duty cycle is fixed over variation in period, the burst duration in this model increases via spike addition. As such, the cycle period varies linearly with spike number (Fig. 5).

The period of a metachronal wave was well approximated by the intrinsic period of the individual oscillator on which that network instantiation was based. For parameter sets that produced activity with higher periods of bursting, the temporal characteristics of individual oscillators better predicted the temporal characteristics of network activity. The metachronal-wave pattern was maintained over a five-fold change in the period of activity.

## Discussion

The flexibility of the central nervous system relies on the ability of neurons to meet a breadth of temporal specifications. Oscillatory neuronal networks, such as central pattern generators, maintain functional output over a wide range of cycle periods in order to produce appropriate behavior [8]–[10], [13]–[24], [75]. Neuromodulation provides control over regimes of activity according to motor tasks by coordinating adjustments to the biophysical parameters of ionic currents [28], [29]. To understand this process, we must answer a key question in neuroscience. How do biophysical characteristics govern functional activity? This question is particularly important for understanding motor control of rhythmic movements. Among the biophysical characteristics that affect excitability, the maximal conductances and voltages of half-activation of ionic currents are the most prominent targets for neuromodulation [25], [26], [61]–[68], [76]–[81].

The maximal conductances of ionic currents are coregulated to produce functional activity [63], [64], [78]–[80]. Homeostasis of a functional pattern of activity, either activity-dependent or activity-independent, has been well documented in terms of covariation of maximal conductances. One mechanism for activity-independent homeostasis of functional activity is determined by the pattern of gene expression of ionic channels. The activity of an identified neuron is specified by the patterns of gene expression of a set of ionic channels [63], [78]–[80]. Correlations have been shown in the quantities of mRNA that code for various voltage gated ion channels in crab [78], [79]. Modeling studies have explored the role of correlation in biophysical parameters in the maintenance of functional activity [60], [82], [83]. The biophysical parameters of hyperpolarization-activated currents and potassium currents are often correlated [63], [78], [79]. For example, coregulation has been revealed by the injection of the mRNA coding A-type potassium current which induced a corresponding increase in 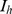 [63]. In most cells considered, the mRNA quantities for these currents are strongly correlated [78], [79]. The mRNA correlations map to correlations in expression of channel protein, which in turn determines the maximal conductance of ionic currents. Correlations in the maximal conductance that support functional activity has been shown in models [60], [82], [83]. Maximal conductances are also correlated in mechanisms of activity-dependent homeostasis [84], [85].

The voltages of half-activation of ionic conductances are also subject to neuromodulation [61], [62], [65]–[68]. These parameters are shifted in neuromodulation of potassium currents and hyperpolarization-activated currents [61], [62], [65]–[68]. For Kv2.1 expressed in HEK293, the voltage dependencies of activation and inactivation are shown to be shifted negatively by 30 mV and 22 mV in response to phosphorylation by AMPK [67]. In the pyloric network of the spiny lobster, depending on the time of exposure and concentration of dopamine, the magnitude and direction of shifts varies but in all cases the shifts appear to be small [65], [66]. Hour-long application of dopamine induces dose-dependent shifts in the voltage dependence of the conductance of an A-type potassium current [66]. In LP neurons, nM and 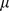
M concentrations positively shift the activation by 4.6 mV and 1.7 mV, respectively, while the shifts of the inactivation curve have different directions: 3.3 mV and −1.2 mV, respectively. In PD neurons, both nM and 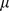
M concentrations negatively shift the activation and inactivation curves by 1.3 mV or less [66]; moreover, a ten minute application of a larger concentration of dopamine (100 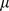
M) induces a larger negative shift of 7.6 mV to the activation curve [65]. In ferret thalamocortical neurons, repeated negative pulses depolarize the activation of 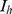 by 3.7 mV, and application of cAMP depolarizes the activation of 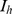 by 12 mV [61], [62]. Neuromodulators can coregulate currents that have complementary effects on membrane dynamics–such as hyperpolarization-activated currents and potassium currents–to tune aspects of excitability [68]. These shifts appear to be small and negligible. However, our results show that precise control of these biophysical parameters in these reported ranges could be sufficient for effective neuromodulation. Amendola et al. [68] show independence of neuromodulation of half-activation of h- current and half-inactivation of A-type potassium current. Such ability to independently control these parameters supports the biological feasibility of the mechanisms of independent control of burst duration and interburst interval by variation of half- activations of 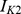 and 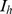, presented in this report.

We have demonstrated a family of cellular mechanisms that provides three different kinds of control for temporal characteristics in neuronal activity by coregulating the kinetics of activation of a non-inactivating potassium current and a hyperpolarization-activated current. These controlling mechanisms are organized by a global codimension-2 bifurcation: the cornerstone bifurcation. The cornerstone bifurcation occurs at the intersection of two codimension-1 bifurcation curves–the saddle-node bifurcation for periodic orbits and the saddle-node bifurcation for equilibria–in the two-dimensional parameter space defined by the voltages of half-activation of 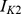 and 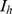.

In our model, the kinetics of activation of 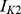 and 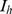 have distinctly separate, complementary roles in rhythmogenesis. 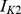, with its voltage of half-activation between 0.006 and 0.011 V (Fig. 1), activates incrementally during each spike in the burst. For this mechanism to function, the activation variable must be significantly slower than the time scale of spiking. Over the course of the burst, this activation accumulates until the current is sufficiently activated to terminate oscillations in the fast subsystem. As the parameter 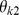 is changed such as to approach the critical value for bifurcation, the steady-state activation curve of 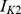 is shifted so that the current is less activated by each spike. Finally, at the critical value for bifurcation, the activation of this current no longer accumulates sufficiently over the course of spiking activity to terminate a burst. 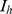 plays a similar role during the interburst interval. Its voltage of half-activation lies roughly between −0.038 and −0.042 V (Fig. 1), and it is responsible for the slow depolarization leading to the first spike in the burst. As the parameter 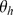 approaches its critical value for bifurcation, the steady state activation curve of 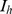 is shifted so that the current is less involved during the interburst interval. At the critical value for bifurcation, this current does not sufficiently activate to initiate the depolarization of the burst.

### Bifurcation Theory and Control of Neuronal Activity

Bifurcation theory considers transitions (bifurcations)–either continuous and smooth or discontinuous and catastrophic–in response to a smooth change in a physical parameter. Applications of bifurcation theory in physics, engineering, and neuroscience have established a new interdisciplinary field: bifurcation control [1], [86], [87]. Bifurcation control offers techniques to control regimes of activity using knowledge of bifurcation locations in parameter space and techniques to control the type of a bifurcation. These techniques prescribe how to precisely control the target characteristics of dynamical regimes by changing bifurcation parameters. Common targets of control include steady state characteristics of equilibria, temporal characteristics and magnitude of oscillatory regimes and transient activity, the borders of basins of attraction, and properties of and the routes to turbulence in deterministic systems [2], [3], [6], [11], [12], [31]–[40], [42]–[50], [87]. For example in the control of turbulent activity, different types of intermittency have been associated with specific bifurcation types [86], [88]. Intermittency of type I and type II are related to the saddle-node bifurcation and the Andronov-Hopf bifurcation, respectively [86], [88].

Bifurcation theory has been applied to describe the general laws of neuronal dynamics that govern characteristics of spiking and bursting activity as a controlling bifurcation parameter approaches a transition between qualitatively different regimes of activity [2], [3], [6], [11], [12], [31]–[40], [42]–[50], [87]. Excitability in neuronal systems has been characterized by type of bifurcation. A saddle-node bifurcation on an invariant circle generates class I excitability [31], [32], [43], [87]. At this bifurcation, a neuron makes a smooth transition from silence into tonic spiking as the bifurcation parameter smoothly changes. This bifurcation controls the interspike interval according to the inverse-square-root law. At the bifurcation point the neuron is silent and, thus, the frequency is zero. The spiking orbit appears at the bifurcation with a large, full-scale amplitude. It is well described by a canonical model: the 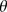 neuron [31], [32], [89]. A supercritical Andronov-Hopf bifurcation generates class II excitability [31], [32], [39], [43]. At this bifurcation, the rest state loses stability, and a spiking orbit with zero amplitude and non-zero frequency is born. The amplitude of spiking grows according to the square root of the bifurcation parameter. Depending on the class of excitability, the response of the neuron to a stimulus, in type 2, may advance or delay the next spike depending on phase or, in type 1, will advance in response to depolarizing pulses and delay in response to hyperpolarizing pulses [31], [32]. The type of phase response describes synchronization in neuronal networks [31], [32], [43]. The quantitative laws described by bifurcation theory are generic and are not uniquely associated with specific biophysical properties of ionic currents. For example, similar dynamical mechanisms may appear in neuronal systems relying on distinct ionic currents [90], and qualitatively distinct dynamics such as type 1 versus type 2 can be realized in a neuronal system by variation of a few biophysical parameters [87]. Dynamical mechanisms have also been used to explain the generation of bursting in terms of slow-fast systems [35], [49], [87]. Bursting is usually based on bistability of spiking activity and a subthreshold regime in the fast subsystem and dynamics of a slow variable governing switches between these regimes [35], [49], [89]. The bursting activity in our model is a square-wave burster [45], [87].

Similarly to spiking activity, temporal laws have been described for bursting activity where the length of the burst duration or interburst interval is subject to control by a bifurcation parameter [11], [12], [42], [45], [48]. The control of a specific temporal characteristic has been shown by the manipulation of single bifurcation parameter. In models of the leech heart interneuron under various experimental conditions, we have extensively studied how bifurcations determine the mechanisms that support bursting activity. Where the blue sky catastrophe controls the transition from bursting to tonic spiking, it imposes the inverse-square-root law on burst duration [45]. Where homoclinic bifurcations control the transition from bursting to the spiking regime–such as the Lukyanov-Shilnikov scenario–a logarithmic law is imposed on burst duration [47]. In this report, we describe independent control of burst duration and interburst interval with two parameters.

Examples of control over burst duration and interburst interval have been shown in a model of the electrosensory lateral line lobe pyramidal cell [11]. In [11], the model shows a unique type of bursting called ghostbursting. In slow-fast decomposition, the fast subsystem of the ghostbursting model does not exhibit bistability [11]; such bistability is a common feature of bursting models [35]. This lack of bistability is described as unique to the ghostbursting mechanism, and as such, it makes a key difference between ghostbursting and our model in the topology of the manifolds that govern slow motion. In our model, bursting activity is based on bistability of spiking and a stable equilibrium in the fast subsystem, thus classified as square-wave bursting; the slow manifolds of spiking activity and equilibria are separated in the phase space. This bistability of regimes in the fast subsystem allows for the bifurcation curves obtained in the full system–the saddle-node bifurcation for periodic orbits and saddle-node bifurcation for equilibria–to cross in the two parameter space. This feature of the bifurcation curves allows the cornerstone bifurcation to generate a family of mechanisms that independently control the temporal characteristics of bursting and transient responses to external stimuli. The curves for saddle-node bifurcation of periodic orbits and saddle-node bifurcation for equilibria cross, dividing parameter space into four regions: spiking, bursting, silence, and bistability of spiking and silence. Similarly to our model, the scenario presented in [11] involves a saddle-node bifurcation for fixed points (SNFP) and a saddle-node bifurcation for periodic orbits (SNLC). In contrast to our model, on the two-dimensional bifurcation diagram in [11], SNLC terminates at SNFP. It is important to emphasize that the saddle-node bifurcation for periodic orbits and the saddle-node bifurcation for equilibria are local bifurcations. In our model, the control of bursting activity is governed by specific global bifurcations: the blue sky catastrophe and the saddle-node bifurcation on an invariant circle are global bifurcations, which include the saddle-node bifurcation for periodic orbits and the saddle-node bifurcation for equilibria, locally. In our model, the two mechanisms, by which burst duration and interburst interval of periodic bursting activity are controlled, are uncoupled.

Another key feature that distinguishes ghostbursting from our scenario, is that in the ghostbursting model the SNLC describes the transition from tonic spiking to chaotic bursting, and SNFP describes the transition of chaotic bursting into silence. Doiron et al. show that these transitions to chaos are intermittency type I [11], [86], [88]. This bursting activity is governed by inverse-square-root laws for burst duration and interburst interval on average. In contrast, our model shows periodic bursting; the blue-sky catastrophe describes the transition between periodic bursting and tonic spiking [45], and SNIC describes the transition between periodic bursting and silence. The ability to control temporal characteristics of periodic bursting activity is critical for the control of precise rhythmic movements. For the first time, a biophysically realistic neuronal model is described which combines two inverse-square-root laws and realizes independent control over burst duration and interburst interval. Moreover, this is the first model of a physical system showing the cornerstone bifurcation. By coordinating burst duration and interburst interval, the duty cycle could be preserved over a wide range of cycle periods under neuromodulatory control.

### A Cellular Mechanism of Pattern Scaling

We suggest that these cellular mechanisms could contribute to control of various types of motor activities. For example, phase shifts of a metachronal-wave pattern could be determined by the duty cycle of each element in a chain of inhibitory coupled bursting neurons. Metachronal-wave patterns are ubiquitous in animal locomotion such as that of the leech, the crayfish, *Drosophila* larvae, and the lamprey [15], [16], [19]–[24]. In each case, the motor pattern is maintained across a range of periods. The lamprey can swim with one body wave cycle with period in the range from 0.13 s to 0.66 s; the leech can swim with one body wave cycle with period from 0.39 to 1.1 s; or *Drosophila* larvae can crawl with body contractions of cycle period from 0.6 s to 1.3 s [13], [15], [16], [23], [24]. Phase lags between spinal segments are scaled such that the lag between neighboring segments is approximately 1% of the cycle period in lamprey, 5% of the cycle period in leech, or 10% of the cycle in *Drosophila* [13], [15]–[17], [19]–[21], [23], [24]. Coupled oscillators have been commonly used in the study of central pattern generators. Phase delay in oscillating patterns have been explained with various network mechanisms such as frequency gradient, coupling gradient, and coding phase delays through adjustment of coupling [18], [34], [52]–[54], [56], [58], [59], [91]. For example, by tuning the coupling strengths and synaptic delays in neuronal networks, complex activity patterns can be generated, stored, and retrieved [58], [59].

*Drosophila* larvae crawl by means of peristaltic contractions of body wall muscles. Posterior-to-anterior waves of contraction propel the animal forward at varying speeds. The segmental phase delay of the propagation of these waves is proportional to the cycle period of oscillations [24]. Fictive motor patterns persist in the isolated nerve cord [19], [20]. The period of this fictive pattern occurs on a time scale of tens of seconds [20], [21]. The phase delay measured from segment to segment in segmental nerve cords is 10% of the period of the motor pattern [20].

We modeled this phenomenon in a chain of coupled bursting neurons. The model corresponded to segments three through seven of the larval *Drosophila* (see Methods). By preserving duty cycle and varying cycle period we achieved maintenance of delay in phase between neighboring neurons in the chain. Moreover, the duty cycle of a model of a single cell predicts the phase delay in the network. Our model demonstrated a five-fold scaling of the metachronal-wave pattern, which is comparable to the two-fold scaling of the motor pattern in *Drosophila* [24]. This is an example of a cellular mechanism which translates to network activity. Although this mechanism does not require extensive synaptic tuning, it could work in conjunction with synaptic mechanisms to produce more a more sophisticated phase delay-cycle period relation.

### Transient Response to Stimulus

Dynamical mechanisms govern rhythmic neuronal activity in the vicinity of bifurcations. These types of mechanisms are often discussed within the context of steady state activity. Stereotyped transient responses and activity could process or represent sensory stimulation [1], [4]–[6], [11], [12], [92]. Such transient responses can be represented by heteroclinic connections in the phase space of a neuronal model. Heteroclinic connections can reliably dominate or control transient neuronal activity by drawing trajectories from a large basin of attraction into a specific and functional response to perturbation before relaxing into steady state activity. Strong attraction to slow manifolds makes evoked responses reproducible. This control manifests as a temporally precise response to inhibition.

These parameters provide two types of control over temporal characteristics for two types of transient activity. First, we demonstrate control of the duration of pulse-triggered bursts in a silent neuron. As the voltage of half-activation of 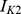 approaches the critical value for the saddle-node bifurcation for periodic orbits, the duration of individual bursts increases. Second, we demonstrated control of the duration of latency to spiking in response to a stimulus in an a periodically spiking neuron. As the voltage of half-activation of 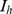 approaches the critical value for the saddle-node bifurcation for equilibria, there is an increase in the latency to spiking after inhibition in periodically spiking neurons. The dynamical mechanisms underlying transient responses described here feature the inverse-square-root law shared with type-I intermittency [11], [12], [86], [88]. In [12], pulses of injected current induced individual transient bursts from a stable periodic spiking regime, called type I burst excitability.

#### Inverse-square-root Law for Pulse-Triggered Burst

In this article, we showed that the silent model could respond to a hyperpolarizing pulse by a stereotypical burst of spikes. We report that the burst duration of the pulse-triggered bursts scaled as 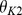 approached the critical value for the saddle node bifurcation for periodic orbits (transition from 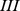 to 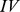). Similarly, Roa et al. have shown the inverse-square-root law in transient spiking activity for type II excitability neuronal models with parameter values near a saddle-node bifurcation for periodic orbits [2]. In Roa et al., a constant applied current is used as the controlling parameter, and an additional brief pulse of current is used to move the phase point away from a stable equilibrium [2]. These models then spend significant time spiking as the phase point passes near the ghost of the saddle-node periodic orbit before relaxing back onto the stable equilibrium. The time neurons spend spiking is proportional to the inverse of the square root of the value of the constant applied current. This mechanism is similar to the inverse-square-root law we reported here for pulse-triggered burst duration with values of 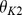 and 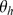 near the border between regions 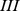 and 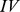. The main difference between underlying mechanisms is that in our model, the phase point moves along a slow motion manifold for oscillations and slows down near the ghost of a saddle-node orbit on that manifold. In our model, the inverse-square-root law in transient activity occurred as the phase point wound around the slow motion manifold.

#### Latency to Spiking After Inhibition

Our model produces a stereotypical latency to spiking after a brief hyperpolarizing pulse in a spiking neuron. By coordinating 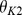 and 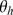, we place the system in a region of the parameter space with a stable tonic spiking regime such that it is close to the saddle-node bifurcation for equilibria (in region 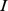 close to the border with region 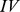). In this region after inhibition, the phase point traveled along the manifold of slow motion and slowed down as it passed the ghost of a saddle-node equilibrium. Control over latency to spiking could explain phenomena crucial for pattern coding and motor control.

In Steuber et al., pauses in Purkinje cell firing are implicated in pattern coding [93]. In their model, a potassium current plays a key role in the mechanism supporting delay to spiking. It is activated by the calcium influx which is induced by sufficient activation of parallel fiber synapses. Pauses in the spontaneous firing of the Purkinje cell occur after parallel fiber-evoked bursts, and long-term depression of parallel fiber synapses shorten pause duration. These pauses code the pattern of synchronous activation of multiple parallel fibers.

Potassium and hyperpolarization-activated currents have been implicated in the dynamics of delay to spiking. In Meng et al., the authors show control over delay to firing by manipulating the ratio of transient potassium conductances [6]. In the brainstem superior paraolivary nucleus, 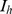 controls the timing of firing in rebound from hyperpolarization [5]. The kinetics of slow conductances may contribute to duration sensitivity after inhibition from a sensory stimulus [4].

### Bistability of Spiking and Silence

Another important feature of the dynamics of our model is that it shows bistability of spiking and silence (region 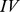, Fig. 3). Perturbations can trigger a switch from one regime to the other (not shown). This phenomenon has been described in the squid giant axon model [39]. In the Hodgkin-Huxley model of the squid giant axon, the stable equilibrium and the oscillating regime are separated by the stable manifold of an unstable orbit [39]. This mechanism was also described in a simplified model of the leech heart interneuron [38]. This model supports a number of types of multistability including a case where a stable equilibrium and a spiking regime co-exist and are separated by the manifold of a saddle equilibrium [38]. Similar mechanisms are described for co-existence of spiking and silence and of bursting and silence in the canonical model of the leech heart interneuron [36], [37]. In the example presented here, the stable periodic orbit sits on the strongly attracting slow manifold for oscillations which is separated from the stable equilibrium by the manifolds of a saddle equilibrium and saddle orbit.

### Designing Artificial Neurons

In the example presented here, a codimension-2 bifurcation that satisfies the criteria for the SNIC and the blue sky catastrophe controls burst duration, interburst interval, pulse triggered bursting, and latency to spiking. In conclusion, we suggest that the control over the temporal characteristics of neuronal activity presented here is critical for designing functional artificial neurons in biomedical and neuroengineering fields [94]. The parameter 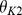 asserts control of burst duration in an endogenously bursting neuron and of duration of pulse-triggered bursts in a silent neuron. The parameter 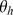 asserts control of interburst interval in an endogenously bursting neuron and latency to spiking in a spiking neuron. By coordinating these parameters, one could tune an artificial neuron to produce bursting activity with any burst duration and interburst interval over a large range of values for the cycle period. Moreover, the transitions between different regimes of activity are smooth and safe over the range of 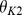 and 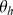 addressed here, so parameters can be tuned without fear of the onset of multistability or catastrophe.

## Methods

We developed a Hodgkin-Huxley style neuronal model. It contains three voltage gated currents: a fast Na^+^ current, 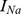; a non-inactivating K^+^ current, 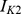; and a hyperpolarization-activated current, 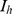. From our previous models, it inherited the blue sky catastrophe [44]–[47]. The model is as follows:
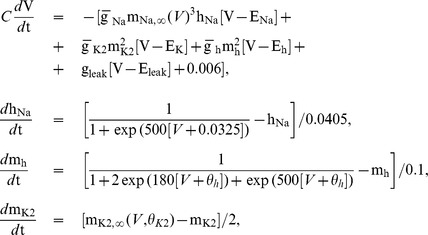
where
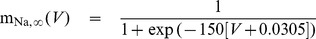
and
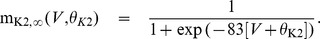



The activation of 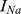 is instantaneous and is denoted as 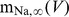. The inactivation of 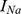 and the activations of 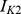 and 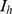 are 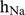, 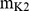, and 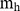. The maximal conductances of ionic currents are 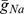 = 105 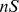, 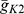 = 30 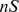, 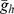 = 4 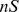, and 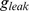 = 8 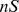. The reversal potentials are 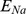 = 0.045 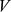, 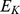 = −0.07 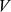, 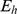 = −0.021 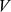, and 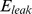 = −0.046 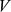. The regulating parameters 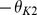 and 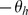 represent the voltages of half-activation of the variables 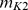 and 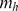. The function 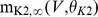 is the steady state activation function of 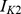. The capacitance, 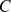, is 2 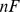. Similar to [45]–[47], 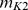 is the slow variable.

The majority of the parameters of this model were pulled directly from our previous model [45]. These models stem from the model explaining the dynamics of slow plateau-like bursting activity in the leech heart interneurons under the conditions when Ca^2+^ currents are blocked with divalent ions and most of the K^+^ currents are blocked with 4-aminopyridine [44], [71], [72]. Here, we added a hyperpolarization activated current as was described in Hill et al. [55]. We adjusted 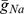 and the time constant of 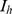 during the search for the cornerstone bifurcation. We also adjusted the time constant of 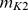, the slow variable, to make the variable slower in order to emphasize the separation of time scales.

We built a model representing the CPG that produces a metachronal-wave pattern of locomotion in *Drosophila* larvae. It follows the generic motif of a chain of coupled oscillators [18], [34], [52]–[54], . This network was composed of a sequence of endogenously bursting model neurons (parameters taken from region 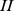 in Fig. 1). We connected these cells into a chain of coupled oscillators, where each node in the chain was an endogenously bursting neuron. We numbered these cells from anterior to posterior according to the segments in the *Drosophila*, such that the most anterior neuron corresponded to the third segment, and the most posterior neuron corresponded to the seventh segment. Coupling was accomplished through inhibitory synapses. The nearest of the posterior cells provided the strongest synaptic input. The synaptic input of the anterior cell was weaker than any of those from posterior cells. The total synaptic current onto cell 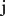 was aggregated into the term 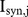, and the current balance equation and expression for synaptic activation became:
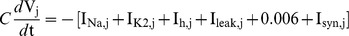


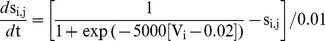



We defined 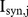 as the sum of the synaptic currents from each presynaptic cell 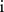 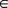
[3 7]:
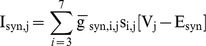


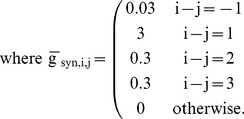



For Figure 4, the synaptic reversal potential was 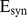 = −0.0625 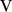. Initial conditions for each cell in the network were almost synchronous. The coordinates were taken from the minimum between the second and third spike in periodic bursting activity. To slightly disturb the synchronous initial conditions, perturbations of 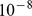 were added to 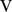 in odd cells. The parameters used to control the cycle period and duty cycle were 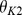 and 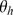 (Table 3).

We performed numerical integration using the 8–9 order Prince-Dormand method from the GNU Scientific Library (http://www.gnu.org/software/gsl/). The continuation of stationary states, periodic orbits, and most bifurcations was performed using CONTENT [95]. The continuation of the saddle-node bifurcation for periodic orbits was performed using XPPAUT [40].

Figure 1 A describes the temporal characteristics of bursting activity at 4294 different parameter values. We sampled the activity on a grid for values of 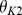 from −0.01054 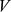 to −0.00602 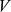 in steps of 0.00004 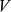 and for values of 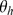 from 0.0375 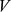 to 0.0413 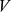 in steps of 0.0001 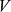.

We analyzed the activity of all trajectories with custom-made scripts in MATLAB (The Mathworks, Inc.). We computed burst duration, interburst interval, cycle period, and duty cycle. Burst duration is the time from the first spike in a burst to the last spike in a burst. Interburst interval is the time from the last spike in a burst to the first spike in the next burst. Cycle period is the time from the first spike in a burst to the first spike in the next burst. Duty cycle is burst duration divided by cycle period.

In Figure S1 and Figure 2, to obtain curve fits to a set of data, we used a Trust-Region optimization routine available in MATLAB. While fitting the expression Eq. 1 (Text S1), the coefficients 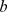, 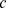, and 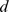 were varied by the optimization routine. While fitting the expression Eq. 2 (Text S1), the coefficients 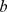 and 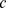 were varied by the optimization routine. The value for 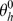 determined in CONTENT and was fixed during optimization. The parameters TolFun and TolX were each 10^−9^.

In Figure S3 and Figure 3, we depict manifolds of slow motion for simple periodic orbits. To identify the blue sky catastrophe, we compared the average coordinate of each orbit to the average value of the nullcline of 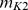 for each orbit on 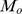. The average coordinate of each orbit was (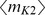, 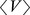) where 
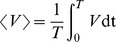
 and 
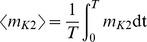
. The average value of the nullcline was (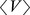, 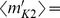 0) where 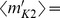 0 is defined by 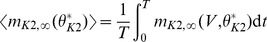. These integrals were computed numerically using the trapezoidal technique with custom-made scripts in MATLAB.

## Supporting Information

Figure S1
**Interburst interval and burst duration are scaled according to saddle-node bifurcations.** Graphs are plotted in the log-log scale. The interburst interval and burst duration are depicted as blue dots. The curves fitted to these data are depicted as red curves. Curve fits for the interburst interval took the form 
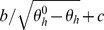
. (A-B) The two examples provided here were computed at fixed values for 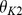 of −0.010 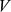 (A) and −0.009 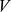 (B) in order to demonstrate that these inverse-square-root laws were general rather than local properties. (A) Coefficients 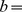 0.00687614 and 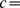 15.85933790. The parameter 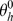 was 0.0413523801025906. (B) Coefficients 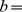 0.00652804 and 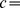 15.10833261. The parameter 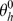 was 0.0413430845706376. (C-D) These examples were provided at values for 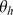 of 0.040 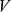 (C) and 0.039 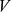 (D). Curve fits for burst duration took the form 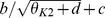. (C) Coefficients 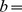 0.97192591, 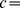 −11.80755281, and 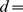 0.01050511. (D) Coefficients 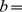 0.97039764 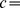 −15.81396058, and 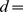 0.01050462.(TIF)

Figure S2
**The dependence of equilibria and periodic orbits on the parameter** 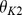 **.** For each orbit, we plot the maximum, minimum, and average voltage. The green curves represent the evolution of a stable orbit as 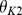 is varied. This stable orbit coalesced with a saddle orbit at a saddle-node bifurcation for periodic orbits (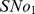). We back-traced this saddle orbit (dashed light blue curves) between 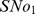 at 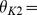 −0.010500 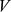 and a second saddle-node bifurcation for periodic orbits (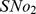) at 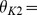 −0.013027 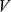 where it coalesced with a stable orbit (solid orange curves). This orbit lost stability in a period doubling bifurcation (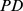) at 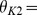 −0.011948 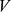. The saddle orbit (dashed dark blue) terminated in a homoclinic bifurcation (Hom). The purple curve represents the equilibria states of the system. The solid purple component indicates a stable equilibrium. The stable equilibria coalesced with the saddle equilibrium (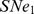) in a saddle-node bifurcation at 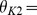 −0.010506 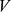 , and this saddle equilibrium coalesces with another saddle equilibrium in a saddle-saddle bifurcation at the point labeled 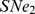 at 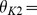 0.029936 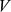.(TIF)

Figure S3
**Structure of the manifolds of slow motion.** (A) The slow motion manifolds for parameter values of both the SNIC and the blue sky catastrophe calculated at 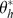. The stable and unstable portions of the slow motion manifold for oscillations are represented by 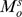 and 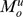, respectively in green and blue. The manifold is composed of many orbits calculated for different values of 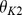 (see Fig. S2). The average voltage is plotted against the average slow variable for each orbit in dark green (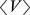). The average nullcline of the slow variable is plotted in orange (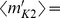 0). The nullcline for the slow variable is represented by the grey curve 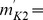 0, and the equilibrium state for the fast subsystem is the purple curve 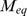. The saddle-node orbit is the closed orange curve labeled as 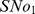. The saddle-node equilibrium is the green dot labeled as 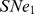. (B) 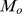 and 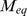 are calculated at 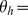 0.038 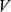. The closed orange curve is a sample periodic burst computed at 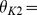 −0.0105 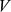 and 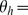 0.038 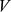. The trajectory of bursting closely follows the manifolds of slow motion.(TIF)

Text S1 **Inverse-Square-Root Curve Fits Confirm SNIC and Blue Sky Catastrophe Bifurcation Curves.**
(PDF)

Text S2 **Computing the Manifolds of Slow Motion.**
(PDF)
