# Supplementary figures and images for: A Codimension-2 Bifurcation Controlling Endogenous Bursting Activity and Pulse-Triggered Responses of a Neuron Model
Source: PLoS One. 2014 Jan 31;9(1):e85451. doi: 10.1371/journal.pone.0085451 (PMC3908860; doi:10.1371/journal.pone.0085451)

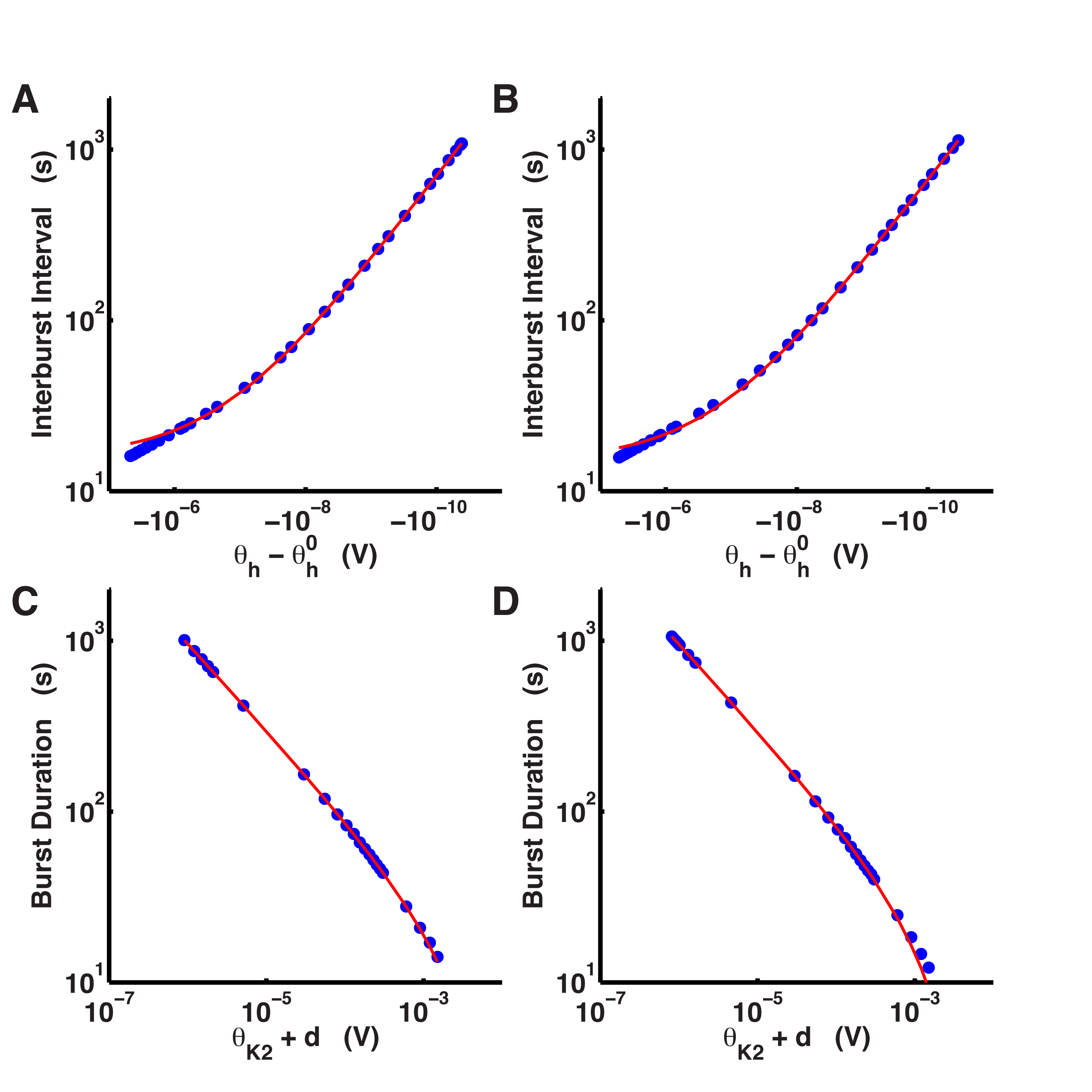

Supplement: Figure S1 — Interburst interval and burst duration are scaled according to saddle-node bifurcations. Graphs are plotted in the log-log scale. The interburst interval and burst duration are depicted as blue dots. The curves fitted to these data are depicted as red curves. Curve fits for the interburst interval took the form . (A-B) The two examples provided here were computed at fixed values for of −0.010 (A) and −0.009 (B) in order to demonstrate that these inverse-square-root laws were general rather than local properties. (A) Coefficients 0.00687614 and 15.85933790. The parameter was 0.0413523801025906. (B) Coefficients 0.00652804 and 15.10833261. The parameter was 0.0413430845706376. (C-D) These examples were provided at values for of 0.040 (C) and 0.039 (D). Curve fits for burst duration took the form . (C) Coefficients 0.97192591, −11.80755281, and 0.01050511. (D) Coefficients 0.97039764 −15.81396058, and 0.01050462. (TIF) [file pone.0085451.s001.tif]

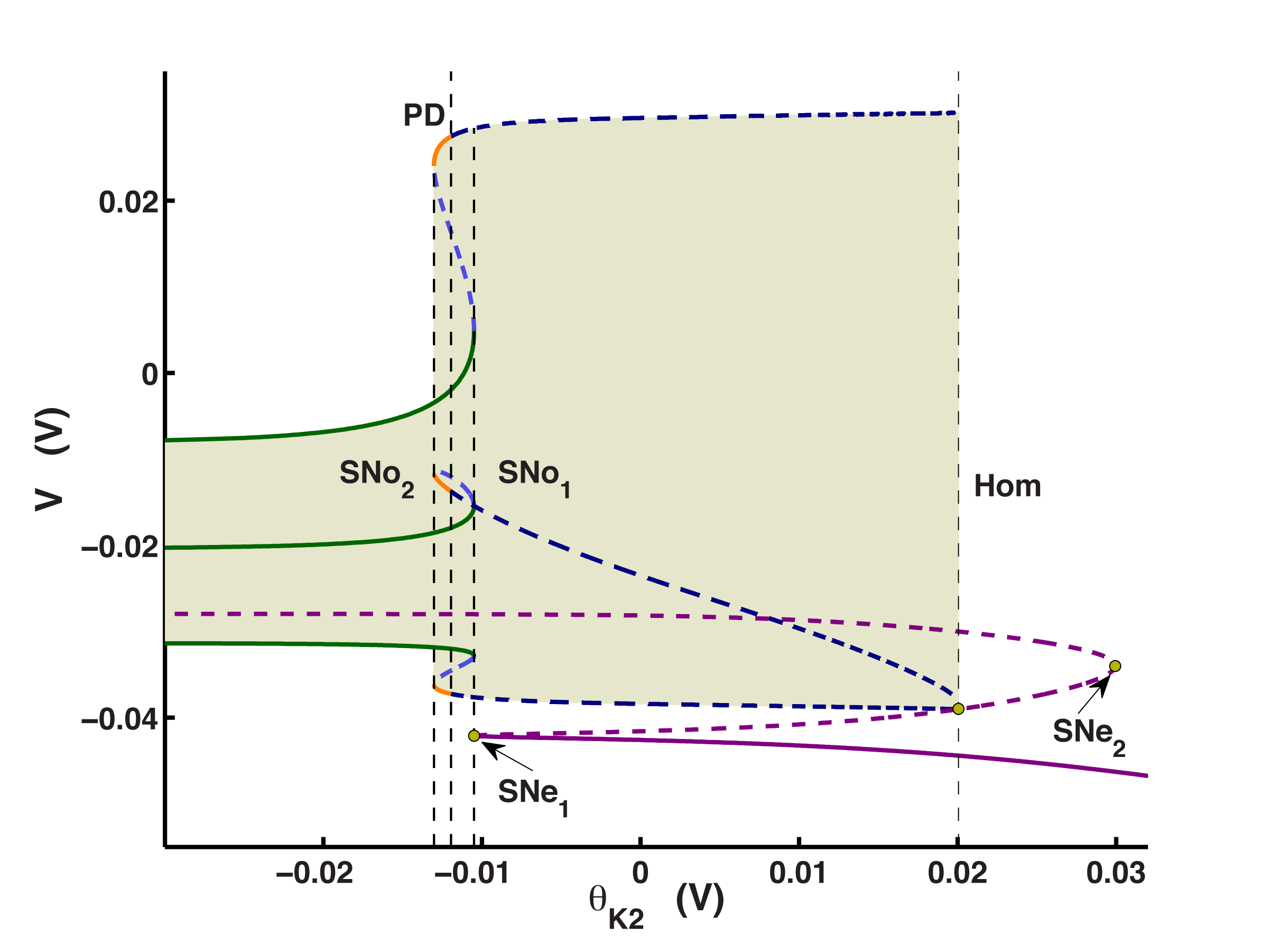

Supplement: Figure S2 — The dependence of equilibria and periodic orbits on the parameter . For each orbit, we plot the maximum, minimum, and average voltage. The green curves represent the evolution of a stable orbit as is varied. This stable orbit coalesced with a saddle orbit at a saddle-node bifurcation for periodic orbits (). We back-traced this saddle orbit (dashed light blue curves) between at −0.010500 and a second saddle-node bifurcation for periodic orbits () at −0.013027 where it coalesced with a stable orbit (solid orange curves). This orbit lost stability in a period doubling bifurcation () at −0.011948 . The saddle orbit (dashed dark blue) terminated in a homoclinic bifurcation (Hom). The purple curve represents the equilibria states of the system. The solid purple component indicates a stable equilibrium. The stable equilibria coalesced with the saddle equilibrium () in a saddle-node bifurcation at −0.010506 , and this saddle equilibrium coalesces with another saddle equilibrium in a saddle-saddle bifurcation at the point labeled at 0.029936 . (TIF) [file pone.0085451.s002.tif]

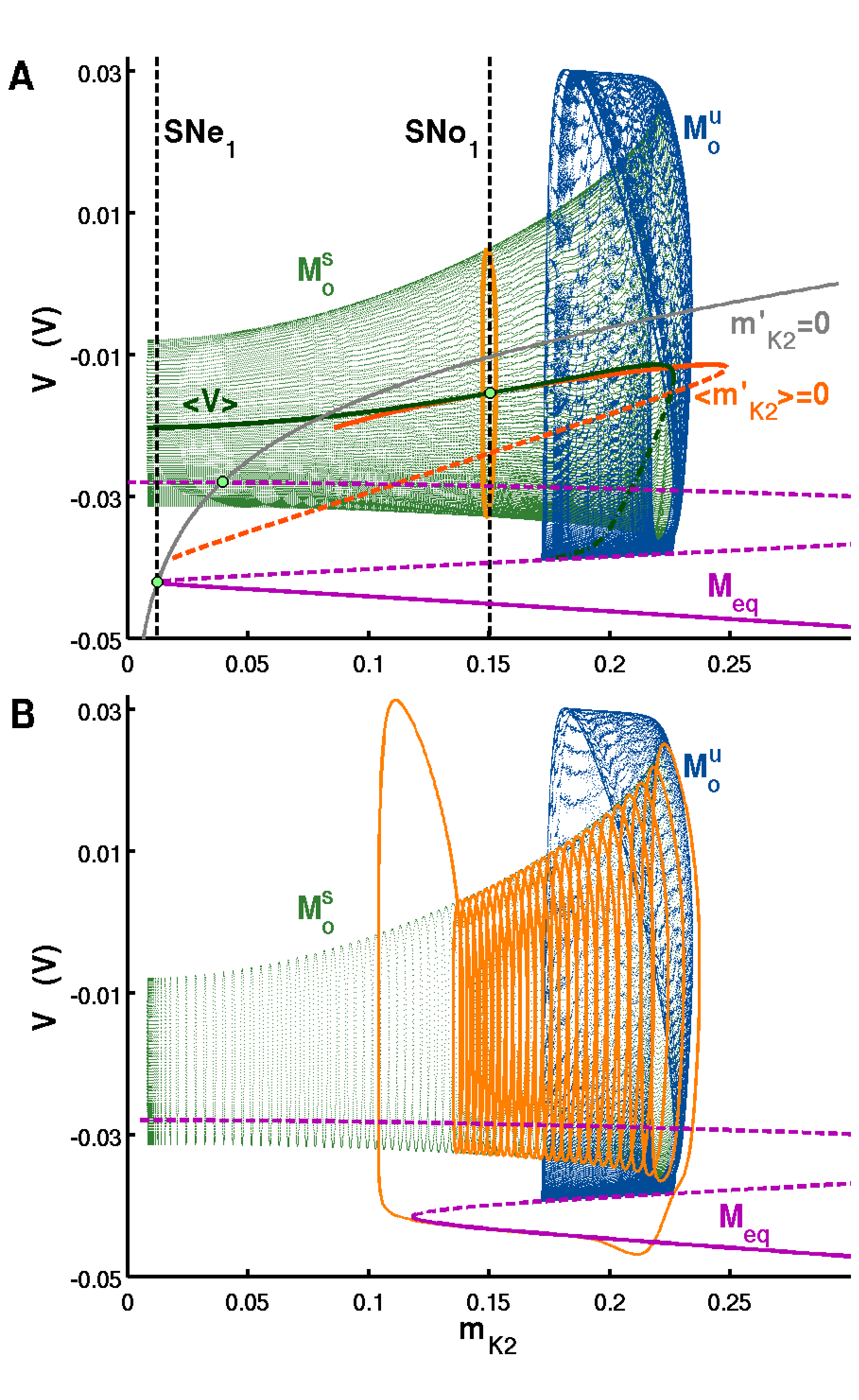

Supplement: Figure S3 — Structure of the manifolds of slow motion. (A) The slow motion manifolds for parameter values of both the SNIC and the blue sky catastrophe calculated at . The stable and unstable portions of the slow motion manifold for oscillations are represented by and , respectively in green and blue. The manifold is composed of many orbits calculated for different values of (see Fig. S2). The average voltage is plotted against the average slow variable for each orbit in dark green (). The average nullcline of the slow variable is plotted in orange ( 0). The nullcline for the slow variable is represented by the grey curve 0, and the equilibrium state for the fast subsystem is the purple curve . The saddle-node orbit is the closed orange curve labeled as . The saddle-node equilibrium is the green dot labeled as . (B) and are calculated at 0.038 . The closed orange curve is a sample periodic burst computed at −0.0105 and 0.038 . The trajectory of bursting closely follows the manifolds of slow motion. (TIF) [file pone.0085451.s003.tif]
