# Supplementary material for: A Codimension-2 Bifurcation Controlling Endogenous Bursting Activity and Pulse-Triggered Responses of a Neuron Model
Source: PLoS One. 2014 Jan 31;9(1):e85451. doi: 10.1371/journal.pone.0085451 (PMC3908860; doi:10.1371/journal.pone.0085451)
Supplement: Text S1 — Inverse-Square-Root Curve Fits Confirm SNIC and Blue Sky Catastrophe Bifurcation Curves. (PDF) [file pone.0085451.s004.pdf]

S1. Inverse-Square-Root Curve Fits Confirm SNIC and Blue Sky Catastrophe Bifurcation Curves

We investigated the temporal characteristics of bursting near these bifurcation curves (Fig S1). The system was directly integrated for a series of parameter values approaching the bifurcation values. We performed curve fits to two slightly different expressions for burst duration and interburst interval:

$$f_{K2}(\theta_{K2}) = b/\sqrt{\theta_{K2} + d} + c, \tag{1}$$

$$f_h(\theta_h) = b/\sqrt{\theta_h^0 - \theta_h} + c. \tag{2}$$

The coefficients b, c, and d were determined with an optimization routine (see Methods for details). Curve fits for burst duration took the form $f_{K2}(\theta_{K2})$ (Eq. 1). Curve fits for the interburst interval took the form $f_h(\theta_h)$ (Eq. 2). The parameter θ_h^0 was the value for the saddle-node bifurcation. We performed curve fits for sixteen data sets – eight distinct data sets for each of the two bifurcations. The dependence of the burst duration and interburst interval on θ_{K2} and θ_h fit well to the quantitative expressions in Eqs 1 and 2, respectively. Qualitatively, the temporal dependence of activity on each parameter corresponds to the analytical prediction of $1/\sqrt{\alpha - \alpha^*}$. As such, these results strongly suggested that the curves depicted in the bifurcation diagram in Figure 1 represent a blue sky catastrophe on the border of the transition from bursting (region II) to spiking (region I) and a SNIC on the border of the transition from bursting (region II) to silence (region III).
