# Supplementary material for: A Codimension-2 Bifurcation Controlling Endogenous Bursting Activity and Pulse-Triggered Responses of a Neuron Model
Source: PLoS One. 2014 Jan 31;9(1):e85451. doi: 10.1371/journal.pone.0085451 (PMC3908860; doi:10.1371/journal.pone.0085451)
Supplement: Text S2 — Computing the Manifolds of Slow Motion. (PDF) [file pone.0085451.s005.pdf]

S2. Computing the Manifolds of Slow Motion

We identified the manifolds of slow motion by analyzing simple periodic orbits and equilibria using the parameter θ_{K2} as a continuation parameter. We produced a family of orbits, M_o, by varying θ_{K2}. Starting at a value (θ_{K2} = -0.03 V) where the system exhibited a stable periodic orbit, we continued this orbit as it went through a series of bifurcations (Fig S2). It folded over twice at saddle-node bifurcations for periodic orbits, first losing stability at SNo_1 and then regaining stability at SNo_2. Shortly after SNo_2, the orbit lost stability in a period doubling bifurcation PD. Also, we computed the slow manifold associated with equilibria, M_{eq}, in the fast subsystem (Fig S2). Starting at a value ($\theta_{K2} = 0.04\ V$) where the system exhibited a stable equilibrium, we continued the equilibrium as it went through two saddle-node bifurcations for equilibria. It lost stability at SNe_1, and folded again at SNe_2. The saddle-node bifurcations at SNo_1 and SNe_1, the two elements of the codimension-2 bifurcation, occurred at the same parameter value. To further confirm that these bifurcations are parts of the cornerstone bifurcation, we show that SNe_1 and SNo_1 occur on the corresponding slow motion manifolds of the full system.

The orbits and equilibria portrayed in Figure S2 are orbits and equilibria of the full system corresponding to different values of θ_{K2}. In addition to portraying these data sets in terms of the bifurcation parameter (Fig S2), we examined each data set in the (m_{K2}, V) projection of the phase space (Fig S3) [1–3]. In this projection, M_o and M_{eq} corresponded to orbits and equilibria in the fast subsystem. Equilibria of the full system are determined by the intersection of the nullcline of the slow variable at a given value of θ_{K2} and M_{eq}. At the critical value for the cornerstone bifurcation, there are two equilibria determined by this analysis: one equilibrium is of the saddle-node type and the other equilibrium is unstable (Fig S3A). SNe_1 was located where the slow manifold, M_{eq}, was tangent to the nullcline of the slow variable (Fig S3A). Orbits of the full system are determined by the intersection of the average nullcline of orbits in M_o evaluated at a given value of θ_{K2} and the average values of V and m_{K2} in M_o. The family of orbits, M_o, was separated into a stable manifold, M_o^s, and an unstable manifold, M_o^u (Fig S3A). Our analysis reduced M_o to the average coordinate of each orbit in M_o: ($\langle m_{K2}\rangle$, $\langle V\rangle$). Additionally, we computed the average value of the nullcline of m_{K2} for each orbit in M_o: ($\langle m'_{K2}\rangle = 0$, $\langle V\rangle$) (see Methods for definitions). We compared the set ($\langle m_{K2}\rangle$, $\langle V\rangle$) to ($\langle m'_{K2}\rangle = 0$, $\langle V\rangle$) to determine the location of orbits of the full system. SNo_1 occurred on M_o^s where the average coordinates of orbits became tangent to the average nullcline of orbits on M_o (Fig S3A). These analyses show that SNo_1 belongs to M_o^s and SNe_1 belongs to M_{eq}.

This structure of the slow motion manifolds produces the dynamics of square-wave bursting (Fig S3B). Our analysis describes the geometry of bursting and the structure of the bifurcation. Bursting trajectories of the full system are located near these manifolds of slow motion [1–4]. Let us describe the motion of the phase point on a bursting trajectory in terms of a slow-fast system. During the burst, the phase point winds fast around M_o^s with m_{K2} slowly increasing. The burst terminates on the fold where M_o^s and M_o^u coalesce. This fold corresponds to SNo_2, which is a saddle-node orbit in the fast subsystem. Following the termination of the burst, the phase point quickly drops onto the stable segment of M_{eq} and follows it with m_{K2} slowly decreasing until SNe_1, which is a saddle-node bifurcation for equilibria in the fast subsystem. The phase point quickly moves up, increasing in V, as the system executes the first spike in a burst and then it is strongly attracted onto M_o^s for the duration of the burst. During the interburst interval, the system follows M_{eq}, and during the burst, the system winds around M_o^s. The direction of these motions is determined by the sign of the derivative of the slow variable (during the interburst interval) or the sign of the averaged derivative of the slow variable (during the burst).

These slow motion manifolds also play a role in the generation of stereotyped transient responses. In a square-wave bursting neuron, the onset of firing and the termination of the burst occur as the system experienced a fast transition from one slow manifold to another. By extracting these features, the evolution of the burst can be broken into two sets of dynamics: those that control the onset of firing and those that control the time course of the burst. In a square-wave bursting neuron, the burst is represented as the winding of the phase point around the slow-motion manifold for oscillations in the

fast subsystem. Similarly, the interburst interval is the period of quiescence that accompanies the slow passage of the phase point over the manifold for equilibria in the fast subsystem. The isolation of these features extends the significance of the slow-fast analysis in endogenously bursting neurons to encompass transient activity in an otherwise silent or spiking cell.

References

1. Shilnikov AL, Cymbalyuk GS (2005) Transition between tonic-spiking and bursting in a neuron model via the blue-sky catastrophe. Phys Rev Lett 94: 048101.

2. Shilnikov AL, Calabrese RL, Cymbalyuk GS (2005) Mechanism of bi-stability: tonic spiking and bursting in a neuron model. Phys Rev E 71: 056214.

3. Cymbalyuk GS, Shilnikov AL (2005) Coexistence of tonic spiking oscillations in a leech neuron model. J Comput Neurosc 18: 255-263.

4. Fenichel N (1979) Geometric singular perturbation theory. J Differ Equations 31: 53-98.
